# A tumor microenvironment-based classification of gastric cancer for more effective diagnosis and treatment

**DOI:** 10.21203/rs.3.rs-3089359/v1

**Published:** 2023-08-01

**Authors:** Dan Duda, Simona Dima, Andrei Sorop, Shuji Kitahara, Namrata Setia, Mihaela Chivu-Economescu, Lilia Matei, Vlad Herlea, Nicolae Pechianu, Takenori Inomata, Aya Matsui, Anna Khachatryan, Shuichi Aoki, Gregory Lauwers, Irinel Popescu

**Affiliations:** Massachusetts General Hospital; Fundeni Clinical Institute; Fundeni Clinical Institute; Tokyo Women’s Medical University; University of Chicago; Stefan S. Nicolau Institute of Virology, Bucharest, Romania Lilia; Stefan S. Nicolau Institute of Virology, Bucharest, Romania; Fundeni Clinical Institute; Fundeni Clinical Institute; Juntendo University Faculty of Medicine; Graduate School of Medical Science, Kanazawa University; Massachusetts General Hospital; Massachusetts General Hospital; H. Lee Moffitt Cancer Center & Research Institute; Fundeni Clinical Institute

## Abstract

With approximately one million diagnosed cases and over 700,000 deaths recorded annually, gastric cancer (GC) is the third most common cause of cancer-related deaths worldwide. GC is a heterogeneous tumor. Thus, optimal management requires biomarkers of prognosis, treatment selection, and treatment response. The Cancer Genome Atlas program sub-classified GC into molecular subtypes, providing a framework for treatment personalization using traditional chemotherapies or biologics. Here, we report a comprehensive study of GC vascular and immune tumor microenvironment (TME)-based on stage and molecular subtypes of the disease and their correlation with outcomes. Using tissues and blood circulating biomarkers and a molecular classification, we identified cancer cell and tumor archetypes, which show that the TME evolves with the disease stage and is a major determinant of prognosis. Moreover, our TME-based subtyping strategy allowed the identification of archetype-specific prognostic biomarkers such as *CDH1*-mutant GC and circulating IL-6 that provided information beyond and independent of TMN staging, MSI status, and consensus molecular subtyping. The results show that integrating molecular subtyping with TME-specific biomarkers could contribute to improved patient prognostication and may provide a basis for treatment stratification, including for contemporary anti-angiogenesis and immunotherapy approaches.

## Introduction

Gastric cancer (GC), with about one million new patients diagnosed yearly, is the third most common cancer with the second highest mortality rate worldwide^1,2^. The vast majority of GCs are adenocarcinomas and can be subdivided into intestinal and diffuse types according to the Lauren classification system^3,4^. The tumor microenvironment (TME), characterized by active angiogenesis, fibrosis, and chronic inflammation, is critical for the local and metastatic progression of malignant solid tumors, including GC^5^. The TME is often characterized by a structurally and functionally abnormal tumor vasculature, which limits drug delivery and suppresses the ability of the immune system to combat malignant cancer^6^. Consequently, chemotherapeutic drugs have limited efficacy in GC, and novel treatment strategies are desperately needed^5^.

Tumor growth depends on angiogenesis – the formation of new vessels – which is essential for solid tumor growth and metastasis^7^. In the absence of vascular growth, tumors cannot develop beyond a few millimeters and therefore remain dormant^8^. The vasculature is a key component of the TME, and its abnormal structure and function mediate tumor progression and treatment responses^9^.

Tumor blood vessels are highly abnormal. They have irregular shapes, diameters, and branching patterns. As such, they cannot be classified as arterioles, venules, or capillaries^10,11^. The consequences of vascular abnormalities in tumors include an aberrant TME, including tissue hypoxia and immunosuppression, and poor drug and effector immune cell infiltration^12^. These abnormalities may contribute to tumor resistance to conventional chemo-, radio-, and immune-based therapies. Jain proposed that judiciously dosed anti-angiogenic treatment can normalize the tumor vasculature by reducing vascular permeability and interstitial fluid pressure, thus improving blood flow and tumor perfusion^13^. A normalized vasculature can reduce hypoxia and enhance the delivery of oxygen and cytotoxic agents for radiation therapy, thereby improving the anti-tumor immune response^14^. Preclinical and clinical studies have supported the hypothesis that anti-angiogenic therapy can normalize the tumor vasculature, at least transiently^5^. Nevertheless, existing classification schemes do not include parameters related to the tumor vasculature or the TME, in part due to the lack of understanding of how the molecular subtype impacts the TME characteristics in GCs^15^.

Malignant cells develop a complex relationship with their TME during progression, which may be a target for enhancing treatment response^16^. Although there is abundant in vitro evidence for immune reactivity against solid tumors in patients, such responses are often ineffective due to local and systemic immunosuppression^17^. Although the immune responses of the host are critical to the success of immunotherapy, such as immune checkpoint inhibition^5^, determinants of the response are not completely understood. Tumor infiltration by immune cells, such as cytotoxic T lymphocytes, varies widely in density, composition, and clinical significance^6,16^. Blood vascular and lymphatic endothelial cells play important roles in the trafficking of immune cells, controlling the microenvironment and modulating the immune response. Improving access to the malignant tumor by altering the vasculature with anti-angiogenic drugs may provide an effective combinatorial strategy for immunotherapy, and might be widely applicable to many tumor types, especially in GC^18^.

Traditional staging systems are important in predicting the prognosis of patients with cancer. These systems are used to stratify patients according to prognostic variables in the setting of clinical trials, allowing the exchange of information among researchers, and finally guiding the therapeutic approach^19^. Although surveillance protocols for patients at risk of developing GC have significantly improved, the clinical outcome remains poor with most patients presenting with advanced disease and not eligible for curative therapy.

At the pathologic level, GC is a morphologically heterogeneous tumor. Despite this knowledge, the staging system used worldwide is based on TMN staging, which has important limitations. Several new classification systems have been proposed to reflect tumor biological diversity, and their implementation may help guide classification, therapy, and biomarker screening for antibody-targeted therapy and immunotherapy^3^.

In this study, we focused on the relationship between our previously described protein-based classifications of GC and the characteristics of their TMEs. This study also examined how a TME-related biomarker approach performs compared to prior approaches such as TMN staging. The results of this study may provide a classification system to facilitate treatment selection, for example for anti-angiogenesis treatment and immunotherapy, which currently benefit only a minority of GC patients. Furthermore, these insights may be useful for designing new treatment approaches against targets in the TME of GC.

## Materials and methods

### Patients

The study included tissues and blood samples from 122 primary GC patients that underwent surgical resection at Fundeni Clinical Institute between 2004 and 2008 and 51 healthy individuals as a control for blood samples. The study was approved by the Ethics Committee of Fundeni Clinical Institute. All the performed experiments were following the declaration of Helsinki and the International Ethical Guidelines for Biomedical Research Involving Human subjects^20^. Collected clinical and pathologic features include age, gender, TNM stage, differentiation degree, tumor size (cm), differentiation degree, lymphovascular and/or venous invasion, perineural invasion, serum tumor biomarker (CA19-9, U/mL), and overall survival (months).

### Tissue analyses

Expression of multiple GC and TME-related biomarkers was measured using EBER in situ hybridization, and immunohistochemistry (IHC) for mismatch repair proteins mucin (MUC)5AC and MUC6. EBER in situ hybridization was the gold standard for detecting Epstein–Barr virus (EBV) status and localizes the abundantly expressed long noncoding RNAs EBER1 or EBER2 in malignant cells ^21,22^. IHC staining for TME markers was performed on 4μm thick sections cut from the representative formalin-fixed, paraffin-embedded tumor tissue. Sections were deparaffinized, and antigen retrieval was performed in a water heater with citrate buffer (pH 9) for CD31, neuron-glial 2 (NG2), carbonic anhydrase 9 (CAIX), VEGFR2, E-cadherin (CDH1), p53 or Tris ⁄ EDTA (pH 9) for programmed death ligand 1 (PD-L1) and CD8 at 97°C. This was followed by 0.03% hydrogen peroxide (H_2_O_2_) treatment for 10 min to block the endogenous peroxidase. Mouse or rabbit monoclonal and polyclonal antibodies against these antibodies (**Supplementary Table 1**) were applied to the sections overnight at 4°C. The slides were then incubated with peroxidase-labeled polymer conjugated to goat anti-rabbit IgG or goat anti-mouse IgG for 30 min. The sections were stained for the appropriate time with DAB, and counterstained with hematoxylin.

### MSI analysis for molecular-based classification

MSI testing was performed using the MSI Analysis System, Version 1.2 kit (Promega), which allows for the analysis of five mononucleotide repeat markers (BAT-25, BAT-26, NR-21, NR-24, and MONO-27) to determine MSI status, as well as two pentanucleotide repeat markers (Penta C and Penta D) used for unique sample identification and to detect potential sample mix-ups or contaminations. For this purpose, 1–2 ng of genomic DNA was subjected to an enzymatic amplification reaction using 1 μl of GoldSTR 10X Buffer and 1 μL of MSI 10X Primer Pair Mix from the kit, and 0.095 μL of Go Taq MDx Hot Start Polymerase (7.9 U/μL), in a final volume of 10 μL. The enzymatic amplification conditions used were: 95°C for 11 min; 96°C for 1 min; 10 cycles of 94°C for 30 sec (ramp up to 58°C at 29% per sec), 58°C for 30 sec (ramp up to 70°C at 23% per sec), and 70°C for 1 min; 20 cycles of 90°C for 30 sec (ramp up to 58°C at 29% per sec), 58°C for 30 sec (ramp up to 70°C at 23% per sec), and 70°C for 1 min; 60°C for 30 min; and 4°C. The resulting reaction products were denatured with a mixture of Hi-Di formamide (Applied Biosystems) and ILS600 weight marker (included in the MSI Analysis System, Version 1.2 kit) by incubation at 95°C for 3 minutes, followed by incubation on ice for 3 minutes, and were separated by capillary electrophoresis using the ABI PRISM^®^ 3130 Genetic Analyzer system (Applied Biosystems). MSI status was determined using GeneMapper software (Applied Biosystems). To determine MSI status, allelic sizes of tumor and normal specimens were compared, and a marker was considered MSI unstable if there was a shift of three base pairs in the cancer samples. Specimens were classified as MSI-High (MSI-H) if two or more microsatellite markers were unstable, MSI-Low (MSI-L) if one marker was unstable, or microsatellite stable (MSS) if no markers were unstable.

### Histopathological scoring

The immunostained sections were scored by evaluating the invasive carcinoma tissue portion. Cytoplasmic and membranous expression of CD31 (endothelial cells), CAIX (hypoxic cells), and NG2 (pericytes) was defined in two groups (low and high) based on the median positive cell surface (%). Microvessel density (MVD) was the surface area covered by CD31-positive cells. Cytoplasmic expression of the VEGFR2 was categorized semiquantitatively based on the percentage of positive cells as follows: 0, no staining; 1, < 10% positive cells stained; 2, 10–25% positive cells stained; and 3, > 25% positive cells stained. Further analyses, we defined VEGFR2 IHC expression in two groups (low and high) based on the median positive cell surface (%). The expression of PD-L1 was only categorized semiquantitatively based on the percentage of positive tumor cells (stroma cells) as follows: 0 (< 1% tumor cells stained); 1(≥ 1% but < 5% tumor cells stained); 2 (≥ 5% but < 10% tumor cells stained); and 3 (≥ 10% tumor cells stained)^23^. In further analyses, we defined PD-L1 IHC expression in two groups based on the percentage of positive tumor cells: low (< 1% cells positive that include IHC score 0) and high (> 1% cells positive, that includes IHC score 1, 2 and 3). Similarly, we defined the IHC expression of infiltrated CD8 lymphocytes in two groups: low (IHC scores 0 and 1) and high (IHC scores 2 and 3) intensity of CD8 positive infiltrate. Cytoplasmic expression of the MUC5AC and MUC6 was categorized semiquantitatively based on the percentage of positive tumor cells as follows: 0, < 10% cells positive; 1, 10–25% cells positive; and 2, > 25% cells positive. To establish a diagnostic value based on mucin expression assessed by IHC, we calculated the average of MUC5AC and MUC6 IHC scores (called MUCavg) for each GC patient. The expression of E-cadherin and p53 have been reported previously^18^. For E-cadherin, the normal expression (presence) was scored with 1, and the aberrant expression (absence) was noted as 0 (**Supplementary Table 1**).

The samples were classified according to the protein expression as previously published^18^, bringing a modification for group 2 (Gp2) by using an MSI analysis that involves comparing allelic profiles of microsatellite markers generated by amplification of DNA from matching normal and tumor samples, which may be mismatch-repair (MMR) deficient. Alleles that were present in the tumor samples but not in corresponding normal samples indicate MSI. The hierarchical clustering resulted in the determination of five groups of gastric adenocarcinomas (Gp1: EBV-positive gastric cancers, Gp2: Microsatellite-instable gastric cancers, Gp3: Gastric cancers with aberrant E-cadherin expression, Gp4: Gastric cancers with aberrant p53 expression, Gp5: Gastric cancers with normal p53 expression).

### Multiplex protein array for circulating biomarkers

We used a chemiluminescence-based multiplexed protein array (MesoScale Discovery) to measure a panel of cytokines/chemokines and angiogenic biomarkers in the serum samples collected from the GC patients in the study (n = 122) and from 51 healthy individuals. All measurements were done in duplicate in a CLIA-certified Core facility at MGH Boston. All samples were obtained from GC patients before surgical resection, thus allowing us to associate the circulating inflammatory factors with tissue biomarkers. The biomarkers panel used for multiplexed protein array analysis in serum samples were angiogenic biomarkers such as basic fibroblast growth factor (bFGF), vascular endothelial growth factor (VEGF)-A, VEGF-C, VEGF-D, soluble (s)TIE2 (TEK receptor tyrosine kinase), placental growth factor (PlGF), soluble VEGFR1 or Fms-like tyrosine kinase-1 (sFLT-1) and inflammatory biomarkers such as interleukin-8 (IL-8), IL-6, tumor necrosis factor alfa (TNF-α) and gamma interferon (IFN-γ).

### Statistical analysis

Quantitative and semiquantitative analyses for tissue markers were performed with the support of experienced MGH gastrointestinal pathologists (GYL and NS). In descriptive statistics, data are presented as n (%) or median (interquartile range (IQR): Q1, Q3). Statistical significance of univariate analysis was determined by the Mann-Whitney-Wilcoxon test with p-values calculated by the exact method and the Kruskal-Wallis test for ordinal or continuous variables with a non-normal distribution. For normal distribution (evaluated with Shapiro test) we used an unpaired t-test with Welch’s correction and one-way analysis of variance (ANOVA), a chi-square test for dichotomous variables followed by Bonferroni post hoc test for multiple comparisons. All P values have been based on 2-sided hypothesis tests. We used log-transformed serum biomarkers to normalize the protein concentration to obtain a normal distribution of data. The Kaplan–Meier method was used to calculate OS curves based on the length of time between primary surgical treatment and final follow-up or death. The log-rank test was used to compare the survival distributions. Differences were considered significant when the p-value was < 0.05. A Cox proportional hazards regression was used for univariate and multivariate analyses of prognostic factors for overall survival. Multivariate survival analysis was performed on variables that were significant in the univariate analyses to identify independent predictors of survival. The hazard ratio (HR) and the corresponding 95% confidence interval (95% CI) were calculated. P < 0.05 was considered statistically significant. Overall survival (OS) was defined as the interval between the date of surgery and the date of death or the end of follow-up. The R packages used in the survival analysis: gtsummary 1.7.1, survival 3.5.5, survminer 0.4.9. The statistical analysis used Graphpad Prism 9.5.1 for Windows (GraphPad Software, Inc) and R 4.2.3 software.

## Results

### Clinicopathological characteristics

This study included 122 patients (34 females and 88 males) diagnosed with resectable GC. The median age was 65 (interquartile range, 57–70 years). The median overall survival (OS) in this cohort was 20 months. These eligible patients signed informed consent and had blood and tissue samples banked. All patients were treated by surgery. Clinical and pathologic parameters are summarized in Table 1. The median age in the control group was 40 (interquartile range, 32–48) years, and distribution by gender was unbalanced with 41 females (80%) and 10 males (20%) (**Supplementary Table 2**).

### Stage-dependent TME characteristics of GCs

**Figure 1:**
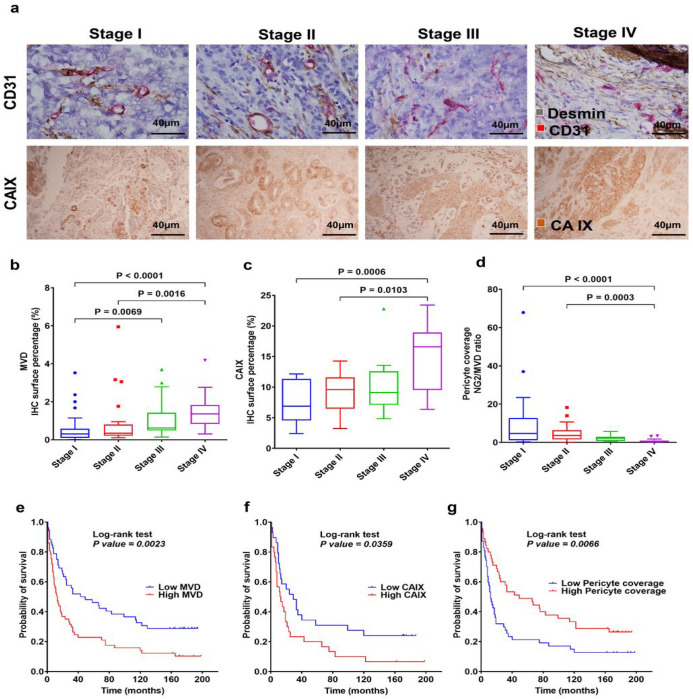
GC progression is associated with increased angiogenesis and vascular abnormalities, the extent of which correlates with worse outcomes. **a** Representative IHC for different stages: CD31+ blood vessels (red) and desmin+ (brown) (first panel); CAIX (second panel). **b** Quantification of CD31+ microvascular density (MVD) showing that it significantly increases with tumor stage (n=110). **c**Quantification of CAIX shows that tissue hypoxia significantly increases with tumor stage (n=59). **d** Quantification of pericyte coverage (NG2/MVD ratio) of GC vessels showing significant decreases in vessel maturation at more advanced GC stages (n=93). **e** The prognostic value of the MVD in patients with GC indicates that the group with high MVD IHC expression had a significantly poorer survival rate (median=13 months) compared to the low MVD IHC expression group (median=46 months). **f** The prognostic value of the CAIX marker in patients with GC shows that the group with high CAIX IHC expression had a significantly poorer survival rate (median=12.5 months) compared to the low CAIX group (median=29 months). **g** The prognostic value of the pericyte coverage in patients with GC. Patients in the low Pc coverage group (median=12 months) have a lower survival rate than the patients in the high Pc coverage group (median=49 months). Comparisons between multiple groups were performed using a Kruskal-Wallis test and correct for multiple comparisons using Dunn’s test. Each box represents the IQR and median of the IHC expression markers levels for each group, whiskers indicate 1.5 times IQR (and any values that are greater than this are plotted as individual points).

The TME-related biomarkers examined in GC surgical samples are summarized in Fig. 1a. MVD was significantly increased in GC tissue from patients with stage IV versus stage I (p < 0.0001) and stage II (p = 0.0016) disease, and stage III versus stage I disease (p = 0.0069) (Fig. 1b). Tissue hypoxia was significantly increased with GC stage, i.e., CAIX expression was higher in stage IV versus stage I (p = 0.0006) and stage II (p = 0.011) disease (Fig. 1c). In addition, pericyte (Pc) coverage of the tumor blood vessels (vessel maturity) in stage IV was significantly decreased compared to stage I (p < 0.0001) and stage II (p = 0.0003) disease (Fig. 1d). These results demonstrate that advanced tumors are associated with more angiogenic (structurally immature) and functionally abnormal vessels in GC. These clinicopathological parameters showed the same trend for association with lymph node status, the presence of metastasis, perineural and lymphovascular invasion, and CA19-9 tumor marker (**Supplementary Fig. 1a-c**). To test the correlation between these tissue biomarkers with outcomes in these GC patients, we used Kaplan-Meier survival distributions and log-rank test, based on median IHC expression of positive cell surface percentage (%), as follows: CAIX low group (< 9.9%) and CAIX high group (≥ 9.9%); MVD low group (< 0.6%) and MVD high group (≥ 0.6%); pericyte coverage (ratio between NG2/MVD IHC positive cell surface percentage): low group (< 1.8 median ratios) and high group (≥ 1.8 median ratios). Kaplan-Meier survival analysis revealed that the groups of patients with high MVD and high CAIX expression had significantly shorter survival compared to the group with low MVD (p = 0.0023) and low CAIX (p = 0.036) (Fig. 1e–f). Moreover, the group of patients with low pericyte coverage had significantly shorter survival compared to the group with high pericyte coverage (p = 0.0066) (Fig. 1g).

### VEGFR2 is expressed across GC subtypes and is associated with tumor and immune TME biomarkers and outcome

**Figure 2:**
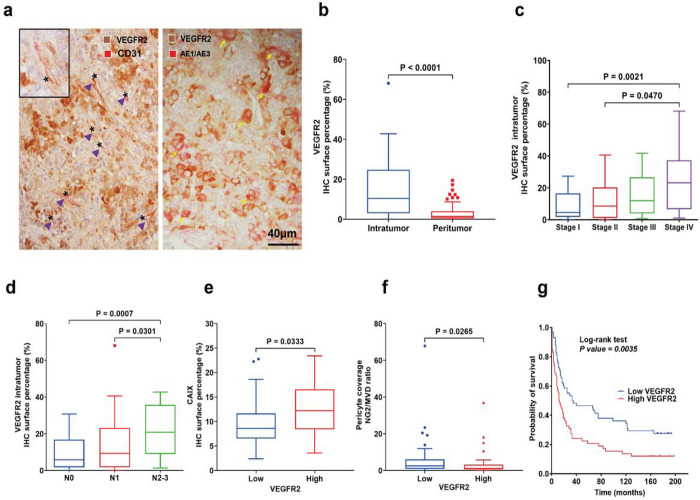
VEGFR2 is highly expressed in GC at all stages and is associated with vascular abnormalities and poor prognosis. **a** Representative immunostaining for VEGFR2 in GC tissue. Both cancer cells and tumor endothelial cells expressed VEGFR2. Asterisks indicate blood vessels. Arrowheads (purple) indicate CD31+/VEGFR2+ endothelial cells. Arrows (yellow) indicate VEGFR2+/AE1/AE3+ tumor cells (n=117). **b** Higher VEGFR2 IHC expression in tumor versus peritumor tissue (n=117). **c** Significant higher intratumor VEGFR2 expression in stage IV versus stage I and stage II (n=117). **d** The IHC expression of VEGFR2 intratumor progressively increases with a higher lymph node stage (n=117). **e** The correlation of CAIX shows significantly increased tissue hypoxia in the high versus the low VEGFR2 expression group (n=58). **f** Significant correlation between low pericyte coverage and high VEGFR2 expression (n=91). **g**Prognostic value of VEGFR2 expression in patients with GC: the group of patients with high VEGFR2 expression had a median OS of 12.5 months versus 34 months in the low VEGFR2 expression group (n=116). Comparisons between multiple groups were performed using a Kruskal-Wallis test and correct for multiple comparisons using Dunn’s test. The difference between the two groups was analyzed by unpaired Mann Whitney test. Each box represents the IQR and median of the IHC expression markers levels for each group, whiskers indicate 1.5 times IQR (and any values that are greater than this are plotted as individual points).

VEGF receptor 2 (VEGFR2) is a validated therapeutic target in advanced GC, based on efficacy data with the antibody ramucirumab. To determine the extent and distribution of VEGFR2 expression in GC, we used double IHC. We detected the expression of VEGFR2 in both endothelial cells (co-localized with CD31 expression), and GC cancer cells (co-localized with cytokeratin expression) (Fig. 2a). VEGFR2 expression was more pronounced intratumorally (median IHC positive cell surface percentage = 10.5%) than in peritumoral tissues (median IHC positive cell surface percentage = 1.4%) (p < 0.0001) (Fig. 2b).

Intratumoral VEGFR2 expression was significantly increased in stage IV versus stage I (p = 0.0021), and stage II disease (p = 0.047) (Fig. 2c). Moreover, expression of VEGFR2 associated with the extent of lymph node metastasis, pN2-3 versus pN0 (p = 0.0007) and pN2-3 versus pN1 (p = 0.030) (Fig. 2d) and with presence of metastasis versus M0 (p = 0.0412) (**Supplementary Fig. 2a**). VEGFR2 expression was not correlated with tumor grading, PNI and LVI (**Supplementary Fig. 2b-d**). In addition, higher VEGFR2 expression was correlated with serum CA19-9 tumor marker level (p = 0.042) (**Supplementary Fig. 2e**).

Furthermore, when comparing the high versus low VEGFR2 expression groups, we found a higher expression of CAIX (p = 0.033) and lower pericyte coverage in the high VEGFR2 expression group (p = 0.026) (Fig. 2e, f). Spearman correlation confirmed that pericyte coverage was inversely correlated with VEGFR2 expression (r=−0.26, p = 0.01), but not with MVD and CAIX expression (**Supplementary Fig. 2f-i**).

When stratified for median VEGFR2 expression (10.5%), Kaplan-Meier analysis showed that the high VEGFR2 expression group had a significantly shorter median OS (12.5 months) versus the low VEGFR2 expression group (34 months) (p = 0.0035) (Fig. 2g).

### Correlation between TME biomarkers and PD-L1 and CD8 T cell infiltration in GC

**Figure 3:**
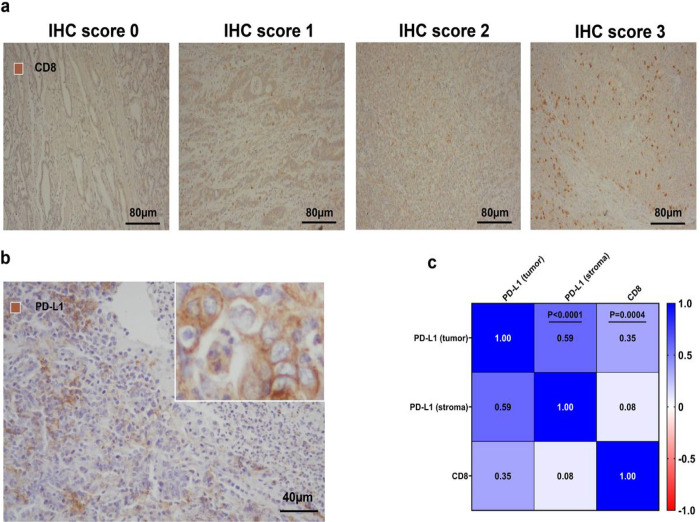
Association between PD-L1 expression and CD8 T cell infiltration in GC tissues. **a-b** Representative IHC for CD8 and PD-L1 in GC tissues. **c** Correlation matrix for PD-L1 and CD8 IHC scores (n=98) (two-sided Spearman’s correlation test).

PD-L1 expression scored by IHC is frequently used for patient selection for immunotherapy. Moreover, The Cancer Genome Atlas (TCGA) project reported elevated PD-L1 expression in EBV-positive GC. Examination of the expression of PD-L1 in the tumor and in the stroma at the tumor periphery and CD8 (to detect this tumor-infiltrating lymphocyte subset) in this cohort by IHC (Fig. 3a, b) revealed that more than 50% of GC cases expressed PD-L1 in cancer and/or in infiltrating immune cells (Table 2). Interestingly, the expression levels of PD-L1 and CD8 in GC tissues were not correlated with tumor stage (**Supplementary Tables 3 and 4**). However, we found that the high PD-L1 expression group had significantly larger tumors compared to the low-expression group (p = 0.012) (**Supplementary Table 3**).

The correlation matrix shows a direct correlation between PD-L1 expression in tumors and CD8 (r = 0.35, p = 0.0004) (Fig. 3c).

Furthermore, the proportion of patients with higher PD-L1 IHC scores in the high CD8 expression group was significantly increased compared with the low CD8 expression group (p = 0.003) (Table 2).

Tian et al. reported that immune responses and vascular normalization are reciprocally regulated in cancer^24^. Thus, we investigated whether PD-L1 and CD8 expression levels in GC were correlated with vascular-related biomarkers such as VEGFR2, MVD, and CAIX IHC expression and pericyte coverage (NG2/MVD ratio). We found that an increased MVD was significantly associated with the high PDL1 expression (p = 0.044) (Table 3). However, VEGFR2, MVD, pericyte coverage, and CAIX were not significantly different between the GC groups with high versus low CD8 expression (**Supplementary Table 5**).

### MUC6 expression associates with pM staging and overall mucin expression with the degree of differentiation

MUC5AC and MUC6 are markers of gastric foveolar and antral/cardiac mucous glandular cells, respectively, reflecting gastric phenotypes. Some prior studies reported correlations between mucin expression and prognosis in GC^25,26^, while others reported conflicting results^27^. We first assessed the IHC expression scores for MUC5AC (**Supplementary Table 6**) and MUC6 (**Supplementary Table 7**) with clinical-pathological data. We found that MUC6 expression only correlated with pM staging (p = 0.029) (**Supplementary Table 7**). In addition, we established an overall expression status of these mucins based on average IHC scores calculated for MUC5AC and MUC6 (called MUCavg) for each GC patient. We defined a low expression group (< 1 MUCavg score) and a high expression group (≥ 1 MUCavg score). The proportion of GCs with well-differentiated status was higher in patients with high MUCavg expression (p = 0.028) (**Supplementary Table 8**). We found no significant associations when testing associations between mucins and TME-biomarkers (**Supplementary Tables 9–12**).

### Circulating angiogenic and pro-inflammatory biomarkers associated with GC and its stage

When comparing the levels of serum biomarkers of angiogenesis in GC patients versus healthy individuals, we found significantly higher levels of bFGF (p = 0.0012), PlGF (p = 0.004), sFLT-1 (p < 0.001), VEGF (p < 0.001), and VEGF-C (p < 0.001) and lower levels of sTIE2 (p < 0.001) and VEGF-D (p = 0.002) (**Supplementary Fig. 3a**). Among inflammatory biomarkers, the circulating levels of IFN-γ, IL-8, and TNF-α were significantly higher in GC patients (p < 0.0001) (**Supplementary Fig. 3b**).

**Figure 4:**
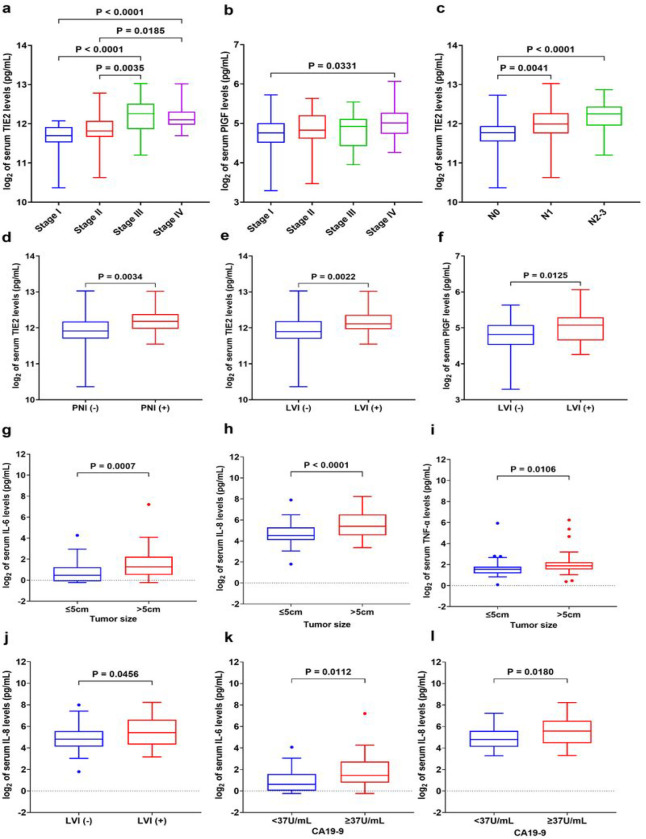
Levels of circulating biomarkers of angiogenesis and inflammation and their correlation with clinical pathological data in GC patients. **a** Serum sTIE2 levels were significantly increased with tumor progression. **b** Serum PIGF levels were significantly higher in stage IV versus stage I. **c** The expression of serum sTIE2 levels progressively increased with the number of metastatic lymph nodules. **d-e** Increased serum sTIE2 levels were significantly correlated with perineural invasion and lymphatic vessel invasion. **f** Serum PIGF levels were significantly increased in GC patients with lymphatic vessel invasion. **g-i** Higher serum IL-6, IL-8, and TNF-α levels in the group of GC patients with larger tumor size (greater than the 5cm median). **j** Serum IL-8 levels directly correlated with lymphatic vessel invasion. **k-l** High levels of serum IL-6 and IL-8 were significantly associated with the serum tumor marker CA19-9. Comparisons between multiple groups were performed using a one-way analysis of variance (ANOVA) with Bonferroni’s post hoc test. The difference between the two groups was analyzed by two-tailed unpaired t-test with Welch’s correction. Each box represents the IQR and median of the normalized serum markers levels for each group, whiskers indicate 1.5 times IQR (and any values that are greater than this are plotted as individual points). Note: expression levels of serum biomarkers were normalized by log_2_.

When we analyzed the association serum between biomarkers of angiogenesis and clinical pathological data, we found significantly higher sTIE2 levels for stage III and IV disease (all p < 0.05) (Fig. 4a) and higher PlGF levels for stage IV versus stage I (p = 0.0331) (Fig. 4b).

Moreover, the levels of sTIE2 directly correlated with the number of pathological metastatic lymph nodes (pN) (Fig. 4c), and sTIE2 levels directly correlated with the presence of perineural invasion (PNI+) and lymphatic vessel invasion (LVI+) (all p < 0.05) (Fig. 4d–e) and serum PlGF levels directly correlated with LVI+ (Fig. 4f). The other angiogenesis biomarkers measured did not correlate with clinical pathological data (**Supplementary Table 13** and **Supplementary Fig. 4**).

When we analyzed the association serum between inflammatory biomarkers and clinical pathological data, we found that patients with GCs larger than the median (> 5 cm) had significantly higher levels of the IL-6 (p = 0.0007), IL-8 (p < 0.0001), and TNF-α (p = 0.011) compared to patients with smaller tumors (≤ 5cm) (Fig. 4g–i). Moreover, serum IL-8 showed significantly higher levels in the group of GC patients with LVI + compared to the group without LVI– (p = 0.046) (Fig. 4j). Interestingly, we found significantly higher expression levels of serum IL-6 (p = 0.011) and IL-8 (p = 0.018) in the GC patients with serum CA19-9 greater than 37U/mL (standard clinical cut-off) (Fig. 4k, l). The other serum proinflammatory biomarkers measured showed no association with the clinical-pathologic characteristics (**Supplementary Table 13** and **Supplementary Fig. 5**).

The TIE2 receptor is expressed on endothelial cells, and together with its ligand angiopoietin (Ang)-1 and Ang-2, plays critical roles in angiogenesis and vessel maturation. The binding of Ang-1 to TIE2 maintains and stabilizes mature vessels by promoting interactions between endothelial cells and the surrounding extracellular matrix^28^.

### Circulating angiogenic and pro-inflammatory biomarkers associated with vascular and immune TME-biomarkers and with OS in GC

**Figure 5:**
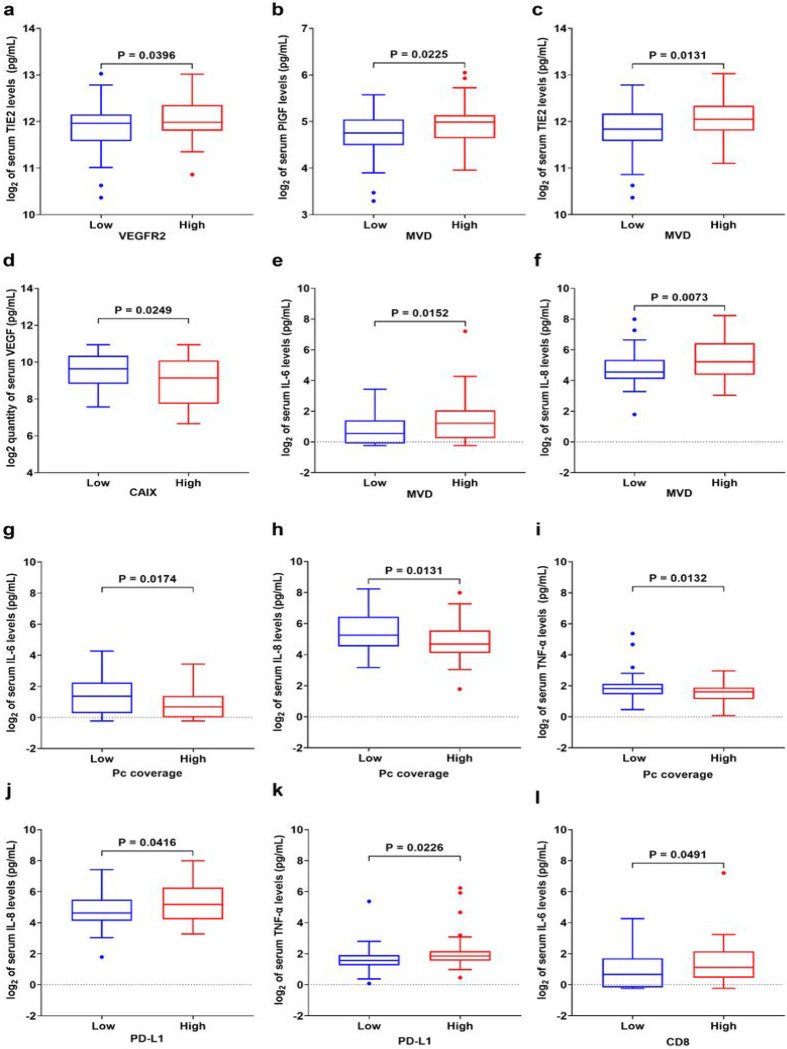
Associations between tissue and circulating biomarkers in GC patients. **a** Correlation between tumor VEGFR2 expression and serum sTIE2 (n=117). **b-c** Correlation between tumor MVD and serum levels of PIGF and sTIE2 (n=110). **d** Correlation between tumor CAIX expression and serum VEGF (n=59). **e-f** Correlation between tumor MVD and serum IL-6 and IL-8 (n=110). **g-i** Correlation between pericyte coverage of GC vessels and serum IL-6, IL-8, and TNF-α (n=93). **j-l** Correlation between tumor PD-L1 expression and circulating levels of IL-8 **(j)** and TNF-α **(k)** (n=116), and between tissue CD8 expression and serum IL-6 (n=101) **(l).** The difference between the two groups was analyzed by two-tailed unpaired t-test with Welch’s correction. Each box represents the IQR and median of the normalized serum markers levels for each group, whiskers indicate 1.5 times IQR (and any values that are greater than this are plotted as individual points). Expression levels of serum markers were normalized by log_2_.

We next tested the associations between the circulating levels and the tissue TME biomarkers, assessed by IHC. We found significant direct correlations between tissue VEGFR2 expression and serum sTIE2 (p = 0.040), between MVD and serum PlGF (p = 0.023), and between MVD and serum sTIE2 (p = 0.013) (Fig. 5a–c). Interestingly, serum VEGF was inversely associated with tissue hypoxia measured by CAIX IHC (p = 0.025) (Fig. 5d). Moreover, MVD was also directly associated with the serum levels of the proinflammatory biomarkers IL-6 (p = 0.015) and IL-8 (p = 0.0073) (Fig. 5e, f). Furthermore, we observed significantly higher expression levels of serum IL-6 (p = 0.017), IL-8 (p = 0.013), and TNF-α (p = 0.013) in the low pericyte coverage group compared to the high pericyte coverage group (Fig. 5g–i). The relationships between the remaining serum angiogenic and proinflammatory biomarkers and the expression of the VEGFR2, MVD, and CAIX markers assessed by IHC did not indicate a significant correlation (**Supplementary Figs. 6 and 7**).

Furthermore, we investigated whether serum biomarkers levels are correlated with PD-L1 and CD8 expression in GC tissues. High PD-L1 expression was directly correlated with serum IL-8 (p = 0.042) and TNF-α (p = 0.023) levels (Fig. 5j, k), and CD8 expression with serum IL-6 levels (p = 0.049) (Fig. 5l). There were no other significant associations between tissue and circulating biomarkers (**Supplementary Fig. 8**).

To examine the diagnostic biomarker significance of serum angiogenic and proinflammatory molecules in resectable GC patients, we established the optimal cut-off value to discriminate between the cancer patients from the control group based on the Youden index and ROC curve analysis (using ‘OptimalCutpoints v1.1.5’ R Package for Computing Optimal Cutpoints in Diagnostic Tests). We found that serum VEGF, VEGFC, sFLT1, sTIE2, IL-8, and TNF-α levels could identify patients with GC based on the optimal cut-off value with AUC > 0.8 (**Supplementary Fig. 9**).

**Figure 6:**
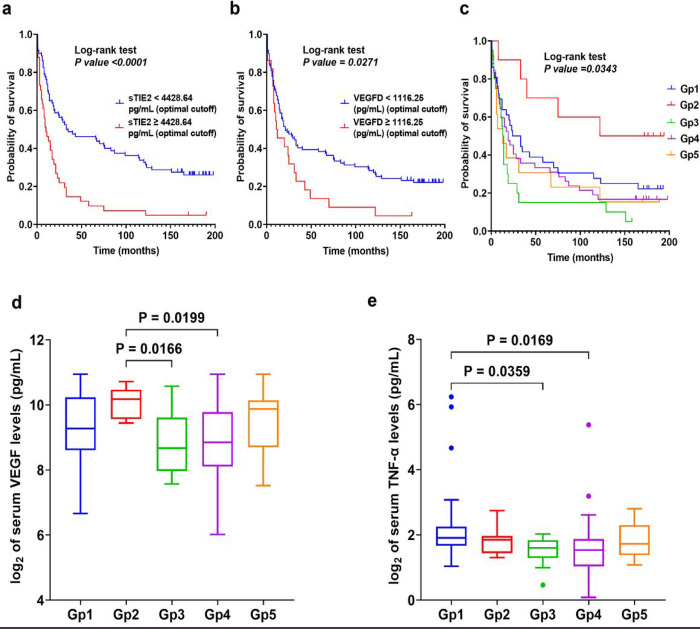
Serum biomarkers association with survival and molecular classes of GC in this cohort (n=122). **a-b** The prognostic value of the serum sTIE2 and VEGF-C in patients with resectable GC Kaplan–Meier curve for overall survival based on molecular classification. **c** Prognostic value of molecular classification. **d** Serum VEGF levels were significantly higher in Gp2 compared to Gp3 and Gp4 groups. **e** TNF-α levels were significantly elevated in Gp1 compared to Gp3 and Gp4 groups. Comparisons between multiple groups were performed using a one-way analysis of variance (ANOVA) with Bonferroni’s post hoc test. Each box represents the IQR and median of the normalized serum markers levels for each group, whiskers indicate 1.5 times IQR (and any values that are greater than this are plotted as individual points). Expression levels of serum markers were normalized by log_2_.

Next, we examined the prognostic biomarker significance of serum angiogenic and proinflammatory molecules in resectable GC patients using optimal cut-off values and the Kaplan-Meier method for OS distributions (n = 121). We found a significantly shorter OS for the GC patients with high (median OS = 10 months) versus low (median = 34 months) serum sTIE2 (4,428.64pg/mL cut-off value; p < 0.0001) and for high (median OS = 11.5 months) versus low (median = 21 months) serum VEGF-D (1,116.25pg/mL cut-off value; p = 0.027) levels (Fig. 6a, b).

Taken together, these findings indicate that serum sTIE2, PlGF, IL-6, IL-8, and TNF-α are biomarkers of tumor progression, vascular abnormalities, and immune suppression in GC.

### Tissue and circulating biomarker association with molecular-based subtypes of GC

We previously reported a classification of GCs in 5 molecular subtypes based on the results of unsupervised hierarchical clustering analysis of the expression EBV in situ hybridization, mismatch repair proteins, E-cadherin, and p53 ^18^. In this cohort (n = 122), 36 GCs were EBER-positive cases (30%) (Gp 1), 10 cases were MSI-high (8.2%) (Gp 2), 20 GCs had E-cadherin deficiency (16%) (Gp 3), and 43 cases (35%) had aberrant p53 expression (Gp 4). The 13 remaining cases comprised 11% of the cases (Gp5) (**Supplementary Table 14**). Among the five groups, age and male predominance were distributed equally, except for Gp2, where the distribution of male/female was equal. TNM staging and median tumor size in each group were not statistically significantly different after the Bonferroni correction (**Supplementary Table 15**). GC patients with Gp2 tumors had a significantly longer median OS (158 months) compared to the other molecular groups (median OS was 28.5 months in Gp1, 13.5 months in Gp3, 19 months in Gp4, and 13 months in Gp5) (p < 0.05) (Fig. 6c and **Supplementary Table 16**).

Next, we examined the levels of tissue and circulating biomarkers in the five GC molecular classes. GCs from the Gp2 class showed non-statistically significant trends for higher PD-L1 expression at the tumor periphery and pericyte coverage of vessels (after the Bonferroni correction test) (**Supplementary Tables 17 and 18**).

Of the serum biomarkers, VEGF was significantly higher in Gp2 compared to the Gp3 (p = 0.017) and Gp4 (p = 0.020) groups, while TNF-α was significantly higher in Gp1 compared to the Gp3 (p = 0.036) and Gp4 (p = 0.017) groups (Fig. 6d, e), with no other difference in other angiogenic (**Supplementary Table 19**) and proinflammatory biomarkers (**Supplementary Table 20**).

### Prognostic significance of clinicopathological parameters, TME- and circulating biomarkers in GC patients

We performed a Cox regression analysis of the association between OS and clinicopathological features, and tissue and blood biomarkers in these GC patients. The univariate Cox proportional hazards regression analysis confirmed the higher risk of death for patients with a higher number of metastatic lymph nodes, pN1 (HR = 2.70, p < 0.001) and pN2 (HR = 9.28, p < 0.001), as well as with more advanced stage: stage III/IV (HR = 3.08, p < 0.001). No association was detected between OS and age, sex, differentiation degree, or tumor size. In addition, a higher risk of death was associated with the molecular groups Gp3 (HR = 4.26, p = 0.004), Gp4 (HR = 2.88, p = 0.027), and Gp5 (HR = 3.28, p = 0.028) versus Gp2 (Table 4 and **Supplementary Table 21**). Univariate analyses of tissue biomarkers identified an association with a higher risk of death for elevated expression of tissue VEGFR2 (HR = 1.83, p = 0.004) and higher MVD (HR = 1.93, p = 0.003) and a non-significant trend for higher MUCavg (HR = 0.67, p = 0.054), (Table 4 and **Supplementary Table 22**). Among the serum biomarkers, a higher risk of death was associated with elevated circulating sTIE2 (HR = 2.13, p = 0.001), IL-6 (HR = 1.35, p < 0.001), and IL-8 (HR = 1.25, p = 0.012) (Table 4 and **Supplementary Table 23**).

Finally, we investigated the significant variables to describe how they correlate with OS. To this end, we performed a multivariate Cox regression analysis, using the proportional hazards assumption for the Cox model using statistical tests and graphical diagnostics based on the scaled Schoenfeld residuals, when including all variables that achieved statistical significance in the univariate analysis (**Supplementary Fig. 10**). The results showed that a higher risk of death was directly associated with pN2 (HR = 13.2, p < 0.001), pN1 (HR = 3.61, p < 0.001), molecular classification Gp3 (HR = 4.28, p = 0.021), and serum IL-6 (HR = 1.40, p = 0.006) (Table 4).

## Discussion

Currently, GC prognostication and treatment selection for immunotherapy is based on clinicopathological features and PD-L1 expression score. In this study, we performed an integrative analysis of tissue and blood biomarkers in GC patients with different disease stages, including a stratification based on molecular classification. This approach adds to previous stage subtyping strategies that have not considered the TME and allowed the development of a model for selecting the targeted therapies in GC (**Supplementary Fig. 11**).

Biomarkers for cancer treatment and diagnosis are defined as biological variables that correlate with biological outcomes, and cancer biomarker discovery strategies that target expressed proteins are becoming increasingly popular^29^. Multiple studies have focused on identifying tumor biomarkers that could facilitate the earlier diagnosis of GC, understanding its behavior, and improving its treatment^30^. However, no single biomarker has been proven predictive of outcomes after antiangiogenic or immunotherapeutic treatments of GC^31^. In addition, few studies have simultaneously evaluated more than one candidate biomarker to enhance the diagnostic sensitivity and specificity of GC^32^. Moreover, there is an increasing interest in developing immunotherapies for earlier GC stages, and many of the current studies are testing combination therapies (anti-angiogenic and immune checkpoint inhibitors, e.g. ClinicalTrials.gov Identifiers: NCT03995017, NCT02443324, NCT04879368, NCT04632459, NCT02999295). Therefore, a logical development to improve the effective diagnosis and treatment of GC is to simultaneously screen for multiple biomarkers, such as TME and immune biomarkers, to increase predictive and response biomarker performance across disease stages.

GCs are inflammation-induced malignancies because they often occur in a diseased stomach on the background of gastritis^33^. This includes the bacterium *Helicobacter pylori* and EBV^34^. These features may be relevant for the immune TME. Indeed, the TCGA project reported elevated PD-L1 expression in approximately 15% of EBV-positive GCs. Evaluation of mRNA revealed elevated expression of JAK2, PD-L1, and PD-L2^1^. In addition, Lin et al. reported that non-Asian GC was significantly enriched in signatures related to T-cell biology, including CTLA-4 signaling. Underlying chronic inflammation and/or viral infections create an immune suppressive TME in GC through the production of cytokines including IL-6, IL-11, TNF-α, and transforming growth factor (TGF)-ß^35,36^. We found that lymph node metastases, pathological stage, molecular classification, VEGFR2 expression by IHC, MVD by IHC, and circulating sTIE2, IL-6, and IL-8 were significantly associated with prognosis. After controlling for the confounding factors, multivariate Cox regression analysis revealed that molecular classification and circulating IL-6 were independent biomarkers of OS.

The infiltration of tumors by immune suppressive leukocytes such as Tregs and myeloid-derived suppressor cells is another important mechanism of immune evasion in cancer. Exhaustion of CD4^+^ T-cells has also been reported as a mechanism of immune evasion in patients with advanced cancer^37,38^. Furthermore, while the immune response to specific antigens is recognized by major histocompatibility receptors, co-stimulatory and co-inhibitory molecules regulate the intensity of the response. Immune checkpoints involve co-inhibitory molecules that are physiologically expressed for the maintenance of self-tolerance^39^. In the cancer microenvironment, including GC, immune checkpoint molecules such as CTLA-4 and PD-L1 are overexpressed and broadly induce the evasive mechanism. Based on these reports, we evaluated the relationship between the expression of PD-L1 and infiltrated CD8^+^ lymphocytes with the tumor microenvironment in GC samples. In our study, PD-1/PD-L1 expression was detected in clinical samples and significantly correlated with tumor progression. We also found that these immune system factors were associated with normalized tumor vasculature and may be important factors in classifying GCs and rationally selecting an effective treatment strategy.

Angiogenesis is a key process in the progression and metastasis of solid malignant tumors. But tumor angiogenesis results in abnormal blood vessels, which create an abnormal TME^40^. Numerous reports revealed that most tumor vessels have deformed diameters, and tumor endothelial cells have loose interconnections, intercellular openings, and abnormal pericytes that are likely to be responsible for vessel leakiness. Moreover, structural abnormalities in the basement membrane of tumor blood vessels are responsible for their relative immaturity compared with normal blood vessels. Accordingly, a tumor blood vessel has abnormal blood flow, is excessively leaky, and has aberrantly high interstitial fluid pressure^9,13,41^. Insufficient perfusion of the tumor tissue in hypovascular areas leads to hypoxia and necrosis. Oncologists and cancer researchers should monitor changes in the aberrant TME for effective treatment and early diagnosis in GC patients^42^. In the current study, we demonstrate that tumor vasculature and TME become increasingly abnormal in advanced stages.

Although MVD is increased in advanced GC, the TME is characterized by hypoxia^5^. Hypoxia may promote GC growth and progression, and resistance to existing therapies. Our study demonstrates that hypoxia is increased in advanced stages of GC. Tumor hypoxia stabilizes HIF1α which induces hypoxia-responsive genes including VEGF. Pathological VEGF expression is a key factor for the abnormal structure and function of tumor vessels^40^. On the other hand, inhibition of the VEGF pathway with approved drugs in advanced GC may result over time and in a dose-dependent manner in excessive pruning of the tumor vasculature, which may also induces hypoxia^43^. Conversely, inducing vessel normalization, alleviating hypoxia, and normalizing the TME might delay GC progression and metastasis^12^.

Thus, we propose that dose titration and identifying the optimal duration of selective anti-VEGF agents are warranted to enhance anti-tumor immunity in GC^6^. This is consistent with our results in advanced liver cancer mouse models^44^ and early-stage clinical studies, including in advanced GC patients^45^. Interestingly, in GC, immunoreactivity for VEGFR2 (the main VEGF receptor responsible for angiogenesis and vascular permeability) was localized to the cytoplasm of endothelial cells but was also present in tumor cells from several histologic subtypes^46^. We also found that VEGFR2 may be useful for classifying GC, diagnosis, and potential for new combination therapy strategies. Future studies should reveal the roles of VEGFR2 on GC cells.

In conclusion, our data show that a TME-related biomarker classification might be potentially useful for existing and new molecularly targeted and immune therapies in GC. Growing evidence is showing that combining anti-angiogenic therapy with immunotherapy may, in certain contexts, improve the immune response in solid cancers^14,47^. A new classification system could also guide more effective new combination therapies, earlier in the management of GC.

## Figures and Tables

**Table 1:** Demographic and clinical-pathological information of gastric cancer patients included in the study

Characteristics	N	N = 122^1^
**Age (years)**	122	
Mean (SD)		63 (10)
Median (IQR)		65 (57, 70)
**Gender**	122	
Female		34 (28%)
Male		88 (72%)
**Staging**	122	
I		30 (25%)
II		31 (25%)
III		31 (25%)
IV		30 (25%)
**Differentiation degree**	122	
Well differentiated (G1)		24 (20%)
Moderately differentiated (G1-G2 and G2)		54 (44%)
Poorly differentiated (G2-G3 and G3)		44 (36%)
**Tumor size (cm)**	122	
Mean (SD)		5.95 (3.02)
Median (IQR)		5.00 (4.00, 7.00)
**Perineural tumoral infiltration (PNI+/-)**	122	
No ( - )		100 (82%)
Yes ( + )		22 (18%)
**Lymphatic vessel invasion (LVI +/-)**	122	
No ( - )		94 (77%)
Yes ( + )		28 (23%)
**Serum tumor biomarker (CA19–9, U/mL)**	97	
Mean (SD)		127 (282)
Median (IQR)		12 (4, 39)
**Overall Survival (months)**	121	
Mean (SD)		58 (68)
Median (IQR)		20 (8, 115)
**Molecular classification**	122	
Gp1: EBV-positive		36 (30%)
Gp2: Microsatellite instability		10 (8.2%)
Gp3: Aberrant E-cadherin expression		20 (16%)
Gp4: Aberrant p53 expression		43 (35%)
Gp5: Normal p53 expression		13 (11 %)

1 Mean (SD), Median (IQR) or Frequency (%)

**Table 2:** Correlation between PD-L1 immune checkpoint (IHC score and IHC expression groups) and CD8 tumor-infiltrating lymphocytes (IHC expression groups)

	Tumor-infiltrating CD8 lymphocytes (IHC expression groups: low = score 0 and 1, high = score 2 and 3)				
Characteristic	N	Overall, N = 102^1^	High, N = 54^1^	Low, N = 48^1^	p-value^2^
PD-L1 IHC score in tumor	98				**0.003** *
0		58 (59%)	25 (48%)	33 (72%)	
1		22 (22%)	12 (23%)	10 (22%)	
2		7 (7.1%)	4 (7.7%)	3 (6.5%)	
3		11 (11 %)	11 (21 %)	0 (0%)	
PD-L1 IHC score in stroma	98				0.3
0		47 (48%)	24 (46%)	23 (50%)	
1		38 (39%)	20 (38%)	18 (39%)	
2		9 (9.2%)	4 (7.7%)	5 (11 %)	
3		4 (4.1%)	4 (7.7%)	0 (0%)	
PD-L1 IHC expression in tumor	98				**0.017** *
Low (IHC score 0)		58 (59%)	25 (48%)	33 (72%)	
High (IHC score 1, 2 and 3)		40 (41 %)	**27 (52%)**	13 (28%)	
PD-L1 IHC expression in stroma	98				0.7
Low (IHC score 0)		47 (48%)	24 (46%)	23 (50%)	
High (IHC score 1, 2 and 3)		51 (52%)	28 (54%)	23 (50%)	

1 Percentage (%)

2 Fisher’s exact test; Pearson’s Chi-squared test; (*, p < 0.05)

**Table 3:** Relationships of PD-L1 IHC expression groups with tumor microenvironment markers

		PD-L1 immune checkpoint (IHC expression groups: low = score 0 and high = score 1, 2 and 3)		
Characteristic	N	Low, N = 63^1^	High, N = 54^1^	p-value^2^
VEGFR2 IHC expression in tumor (%)	114	13 (3, 27)	10 (3, 21)	0.5
VEGFR2 IHC expression in periphery (%)	106	1.1 (0.6, 3.5)	1.6 (0.4, 4.2)	0.9
MVD IHC expression (%)	108	0.51 (0.25, 1.15)	0.80 (0.35, 1.78)	**0.044** *
Pc coverage (ratio)	93	2.2 (0.7, 4.8)	1.3 (0.7, 4.7)	0.6
CAIX IHC expression (%)	59	11.2 (7.5, 15.9)	9.7 (6.5, 12.2)	0.3

1 Median (IQR)

2 Wilcoxon rank sum test; Wilcoxon rank sum exact test; (*, p < 0.05)

**Table 4:** Univariate and multivariate Cox proportional hazards regression analyses of significant variables in association with overall survival of GC patients

	Summary data	Univariate				Multivariate			
Characteristic	N = 122^1^	N	HR^2^	95% CI^2^	p-value	N	HR^2^	95% CI^2^	p-value
**pN (metastatic lymph nodes)**		121				106			
0	36 (30%)		-	-			-	-	
1	49 (40%)		2.70	1.56, 4.68	**< 0.001**		3.61	1.80, 7.28	**< 0.001**
2	37 (30%)		9.28	4.94, 17.4	**< 0.001**		13.2	5.00, 34.8	**< 0.001**
**Staging**		121				106			
I/II	61 (50%)		-	-			-	-	
III/IV	61 (50%)		3.08	2.03, 4.68	< 0.001		0.49	0.24, 1.00	0.051
**Molecular classification**		121				106			
Gp2	10 (8.2%)		-	-			-	-	
Gp1	36 (30%)		2.38	0.92, 6.17	0.074		1.29	0.44, 3.79	0.6
Gp3	20 (16%)		4.26	1.58, 11.5	**0.004**		4.28	1.25, 14.7	**0.021**
Gp4	43 (35%)		2.88	1.13, 7.38	**0.027**		2.27	0.78, 6.58	0.13
Gp5	13 (11 %)		3.28	1.14, 9.45	**0.028**		1.87	0.59, 5.95	0.3
**VEGFR2 expression (IHC)**		116				106			
low	59 (50%)		-	-			-	-	
high	58 (50%)		1.83	1.22, 2.77	**0.004**		1.23	0.78, 1.96	0.4
**MVD (IHC)**		109				106			
low	53 (48%)		-	-			-	-	
high	57 (52%)		1.93	1.26, 2.96	**0.003**		1.27	0.78, 2.05	0.3
**sTIE2 (pg/mL)**		121	2.13	1.34, 3.39	**0.001**	106	1.58	0.83, 3.00	0.2
Mean (SD)	11.97 (0.44)								
**IL-6 (pg/mL)**		121	1.35	1.14, 1.61	**< 0.001**	106	1.40	1.10, 1.77	**0.006**
Mean (SD)	1.07 (1.21)								
**IL-8 (pg/mL)**		121	1.25	1.05, 1.48	**0.012**	106	1.12	0.91, 1.39	0.3
Mean (SD)	5.06 (1.20)								

1 Mean (SD) or Percentage (%)

2 HR = Hazard Ratio, CI = Confidence Interval. Note: concentration values of serum markers were normalized by log_2_

In bold text, p values less than 0.05
